# Author’s reply: The first tick-borne encephalitis case in the Netherlands: reflections and a note of caution

**DOI:** 10.2807/1560-7917.ES.2016.21.39.30356

**Published:** 2016-09-29

**Authors:** Vishal Hira, Joris A de Graaf, Barry Rockx, on behalf of the authors of the original article

**Affiliations:** 1 Department of Medical Microbiology and Immunology, St. Antonius Hospital, Nieuwegein, The Netherlands; 2 Department of Neurology, Zuwe Hofpoort Hospital, Woerden, The Netherlands; 3 National Institute for Public Health and the Environment (RIVM), Bilthoven, The Netherlands; 4 http://www.eurosurveillance.org/ViewArticle.aspx?ArticleId=22558

**Keywords:** The Netherlands, viral infections, tick-borne diseases, tick-borne encephalitis - TBE, viral encephalitis, laboratory, molecular methods

**To the editor:** We thank Clement et al. for their interest in our report on the first tick-borne encephalitis virus (TBEV) infection in the Netherlands (which was acquired in May, but diagnosed in July). They express their concerns about the correctness of the diagnosis and correctly point out several oddities and diagnostic challenges in the case. However, despite these uncertainties, we have no doubt that our patient was infected with TBEV because of several reasons.

Firstly, we did not rely solely on serological techniques, as the patient had provided us with the tick that had bitten him; the species was phenotypically not determinable because it had dried. The tick was TBEV-positive in qRT-PCR, not for the novel Dutch TBEV (a separate manuscript with sequence data of this virus is currently under review), but for a TBEV strain very closely related to the Neudörfl strain, implying the presence of several distinct strains of TBEV in the Netherlands. In our opinion, knowing that the patient had been bitten by a tick proven to be TBEV-infected, drastically increases the chance that compatible symptoms and positive anti-TBEV serology was attributable to a true TBEV infection.

Secondly, according to the paper by Holzmann and the recent European Union case definition for TBE, our patient met the criteria for a proven/confirmed TBEV infection. These criteria are, among others: symptoms of inflammation of the central nervous system and presence of specific IgM and IgG antibodies in blood and/or cerebrospinal fluid (CSF), in the absence of vaccination in the previous months [1,2]. In our case, the specificity of serum antibodies was confirmed with neutralisation tests (NT). Although, based on one study in animals [3], the specificity of NT is disputed, we believe the antibodies in our case were specific because NT titres were high. As the samples (indeed taken on days 24 and 36) were not diluted beyond 1/640 (the goal of the NT was to confirm the specificity of our ELISA results), we do not know the exact titres and thus their dynamics. In the cited animal study, cross-reactivity with louping ill virus was observed and all West Nile virus-infected horses were negative in TBEV NT [3]. As louping ill virus is not known to circulate in the Netherlands, we do not believe this has affected our results. Positive anti-TBEV antibodies may indeed have been due to a yellow fever vaccination 11 years earlier, but this is only true for IgG. IgM is only detectable for several months after vaccination [1]. Furthermore, the substantial decrease in anti-TBEV IgM concentration between days 24 and 36 cannot be explained by this vaccination, but is not unusual in a recent TBEV infection [1].

Thirdly, we should indeed have determined a CSF/serum IgG ratio. We recently determined this ratio and found it to be negative. Intrathecally produced anti-TBEV antibodies are, however, not mandatory for a definite diagnosis. Although the presence of intrathecally produced antibodies is indeed supportive of the diagnosis, they are not found in all cases. Kaise and Holzmann, for example, found that 16% of TBEV-infected patients did not have intrathecally produced anti-TBEV antibodies at hospital admission [4]. More recently, Henningsson et al. described a similar case, also without anti-TBEV antibodies in CSF [5].

In conclusion, we agree that our case had several unusual aspects, such as the predominant mononuclear cell reaction in CSF and the lack of intrathecally produced anti-TBEV antibodies. We believe, however, that our patient had a proven TBEV infection, based on the presence of compatible clinical symptoms, the presence of TBEV-specific antibodies in two different assays and the high levels of TBEV in the tick that had bitten the patient.
